# Expression pattern and prognostic impact of glycoprotein non-metastatic B (GPNMB) in triple-negative breast cancer

**DOI:** 10.1038/s41598-021-91588-3

**Published:** 2021-06-09

**Authors:** Yu-Hsiang Huang, Pei-Yi Chu, Ji-Lin Chen, Chun-Teng Huang, Chi-Cheng Huang, Yi‐Fang Tsai, Yu-Ling Wang, Pei-Ju Lien, Ling-Ming Tseng, Chun-Yu Liu

**Affiliations:** 1 School of Medicine, National Yang Ming Chiao Tung University, Hsinchu, Taiwan; 2 Department of Pathology, Show Chwan Memorial Hospital, Changhua City, Taiwan; 3 School of Medicine, Fu Jen Catholic University, New Taipei City, Taiwan; 4 Comprehensive Breast Health Center, Taipei Veterans General Hospital, Taipei, Taiwan; 5 Division of Medical Oncology, Department of Oncology, Taipei Veterans General Hospital, Taipei, Taiwan; 6 Division of Hematology & Oncology, Department of Medicine, Yang-Ming Branch of Taipei City Hospital, Taipei, Taiwan; 7 Division of General Surgery, Department of Surgery, Taipei Veterans General Hospital, Taipei, Taiwan; 8 Department of Public Health, College of Public Health, National Taiwan University, Taipei, Taiwan; 9 Division of Experimental Surgery, Department of Surgery, Taipei Veterans General Hospital, Taipei, Taiwan; 10 Department of Nursing, Taipei Veterans General Hospital, Taipei, Taiwan; 11 Division of Transfusion Medicine, Department of Medicine, Taipei Veterans General Hospital, No. 201, Sec. 2, Shih-Pai Road, Taipei, 112 Taiwan

**Keywords:** Breast cancer, Prognostic markers

## Abstract

Glycoprotein non-metastatic B (GPNMB) is a transmembrane protein overexpressed in numerous cancers including triple-negative breast cancers (TNBC). It has been linked to promote cancer aggressiveness and implicated as a novel target for GPNMB-expressing cancers. In current study, we aimed to explore the clinical significance of GPNMB in TNBC. Among 759 specimens, immunohistochemistry (IHC) exhibited GPNMB expressions were variable in different subtypes and significantly higher in TNBC. Kaplan–Meier analysis revealed GPNMB overexpression in TNBC was associated with worse prognosis especially distant metastasis (*P* = 0.020, HR = 2.515, CI 1.154–5.480). Multivariate analysis showed GPNMB expression was an independent prognostic factor in terms of recurrence and distant metastasis (*P* = 0.008, HR = 3.22, CI 1.36–7.61; *P* = 0.017, HR = 3.08, CI 1.22–7.74). In silico analysis showed high mRNA expression of GPNMB was associated with distant metastasis and GPNMB was overexpressed in TNBC. Furthermore, GPNMB positively correlated with epithelial–mesenchymal transition (EMT) regulators, mesenchymal marker vimentin, MMP and integrins. The protein levels of Twist and MMP2 were upregulated by GPNMB overexpression in TNBC cells. GPNMB-enhanced cell invasion was attenuated by broad spectrum MMP inhibitor (GM 6001) and the selective inhibitor of MMP-2 (ARP100). In summary, GPNMB expression is prevalent in TNBC and may be implicated as a prognostic biomarker in patients with TNBC.

## Introduction

Triple-negative breast cancer (TNBC), characterized by lack of estrogen receptor (ER), progesterone receptor (PR), and human epidermal growth receptor 2 (HER2) amplification, harbors higher recurrence rate than non-TNBC counterparts^1^. TNBC remains a difficult-to-treat breast cancer subtype for it per se is also heterogenous. Several new therapeutic strategies including antibody–drug conjugate (ADC) approach have emerged as promising ones, highlighting the importance of characterizing expressions of these molecular antigens targeted by ADCs in TNBC^2^. There have been several surface molecules studied as targets for the use of ADCs, including Trop-2^3^, LIV-1^4^ and GPNMB^5^. Since defining a suitable candidate of cell surface markers for ADCs might be challenging, a better understanding in interactions of potential ADC targets provides a greater chance of overall success and a superior medication^6^. There have been successful ADCs approved for other cancers such as trastuzumab emtansine for HER2-amplified breast and gastric cancers^7^, fam-trastuzumab deruxtecan-nxki for metastatic HER2-positive breast cancer^8^, brentuximab vedotin for CD30-positive Hodgkin lymphoma and CD30-expressing peripheral T-cell lymphomas^9^. HER2 plays a distinct role in epidermal growth factor receptor and CD30 involves in tumor necrosis factor signaling, the biological roles of these cell surface markers are important in cell division pathways^6,10,11^. Targeting the cell surface markers involved in cancer cell proliferation and metastasis can be advantageous for ADCs efficacy^6^. GPNMB is a tumor cell surface marker that has the potential to promote tumor growth and invasion.

GPNMB, also known as osteoactivin, was first isolated and discovered in metastatic melanoma in 1995^12,13^. It is a type I transmembrane protein comprised of three domain including extracellular domain, transmembrane domain and cytoplasmic domain^14^. A growing number of studies have shown that GPNMB was expressed in several cancer tissues, such as breast cancer^15^, lung cancer^16^, hepatocellular cancer^17^, stomach cancer^18^, colorectal cancer^19^, prostate cancer^20^, bladder cancer^21^ and glioblastoma multiforme^22^. GPNMB is also known as hematopoietic growth factor inducible neurokinin-1 (HGFIN)^23^ and dendritic cell-associated heparin sulfate proteoglycan-dependent integrin ligand (DC-HIL)^24^. Moreover, higher GPNMB level has been demonstrated to promote angiogenesis, migration, invasion and metastasis of cancer cells^15,25–27^.

Rose et al. has reported a 29% (30 of 103) GPNMB positivity in TNBC as defined as ≥ 5% positively stained tissue on immunohistochemical (IHC) staining, contrast to a 3.5% positivity in normal breast tissue^5^. Moreover, they found that GPNMB expression is associated with poor outcomes in TNBC. Glembatumumab vedotin (CDX-011), an ADC targeting GPNMB, was designed to bind to extracellular domain of GPNMB, and after internalization, release potent cytotoxic agent in patients with metastatic TNBC^28^. Although the precise mechanism of GPNMB underpinning the tumor cells remains unknown, there is an urge to find the therapeutic options for patients with TNBC^29^. In present study, we aim to investigate the clinical and biological significance of GPNMB in patients with TNBC.

## Results

### Patients with TNBC exhibits GPNMB overexpression compared to patients with non-TNBC

Clinical follow-up data were available for 759 patients with primary breast cancer included in the study, with a median follow-up of 74 months. In the cohort of 759 patients, 98 (12.9%) were classified as TNBC. Among the TNBC, the median age of patients was 55.0 years (interquartile range 48.7–66.2 years, Table 1). TNM staging system of the majority of patients were T2 (n = 59, 60.2%), N0 (n = 56, 57.1%) and stage II (n = 55, 56.1%). In terms of lymphovascular invasion, 76 patients (77.6%) were absence of lymphovascular invasion.

**Table 1:** General characteristics of 98 patients with triple-negative breast cancer (TNBC).

Characteristic	n = 98	%
**Age, years (Median, IQR)**^a^	55.0 (48.7 to 66.2)	
**Tumor stage (T)**^b^		
Tis	1	1.0
T1	31	31.6
T2	59	60.2
T3	2	2.0
T4	5	5.1
**Nodal status (N)**		
N0	56	57.1
N1	22	22.4
N2	7	7.1
N3	13	13.3
**AJCC TNM stage**		
DCIS	1	1.0
I	19	19.4
II	55	56.1
III	23	23.5
**Lymphovascular invasion**		
Absent	76	77.6
Present	21	21.4
NA	1	1.0
**Tumor necrosis**		
Absent	41	41.8
Present	57	58.2
**Histologic grade**		
1	5	5.1
2	31	31.6
3	62	63.3
**GPNMB H-score (Median, IQR)**^c^	87.5 (45.0 to 165.0)	

^a^IQR, Interquartile range; DCIS, Ductal carcinoma in situ.

^b^Stage was according to 7th edition of AJCC staging^56^.

^c^Data were expressed as H-score, as described in Method section.

**Figure 1Uploaded the correct version of figure 1:**
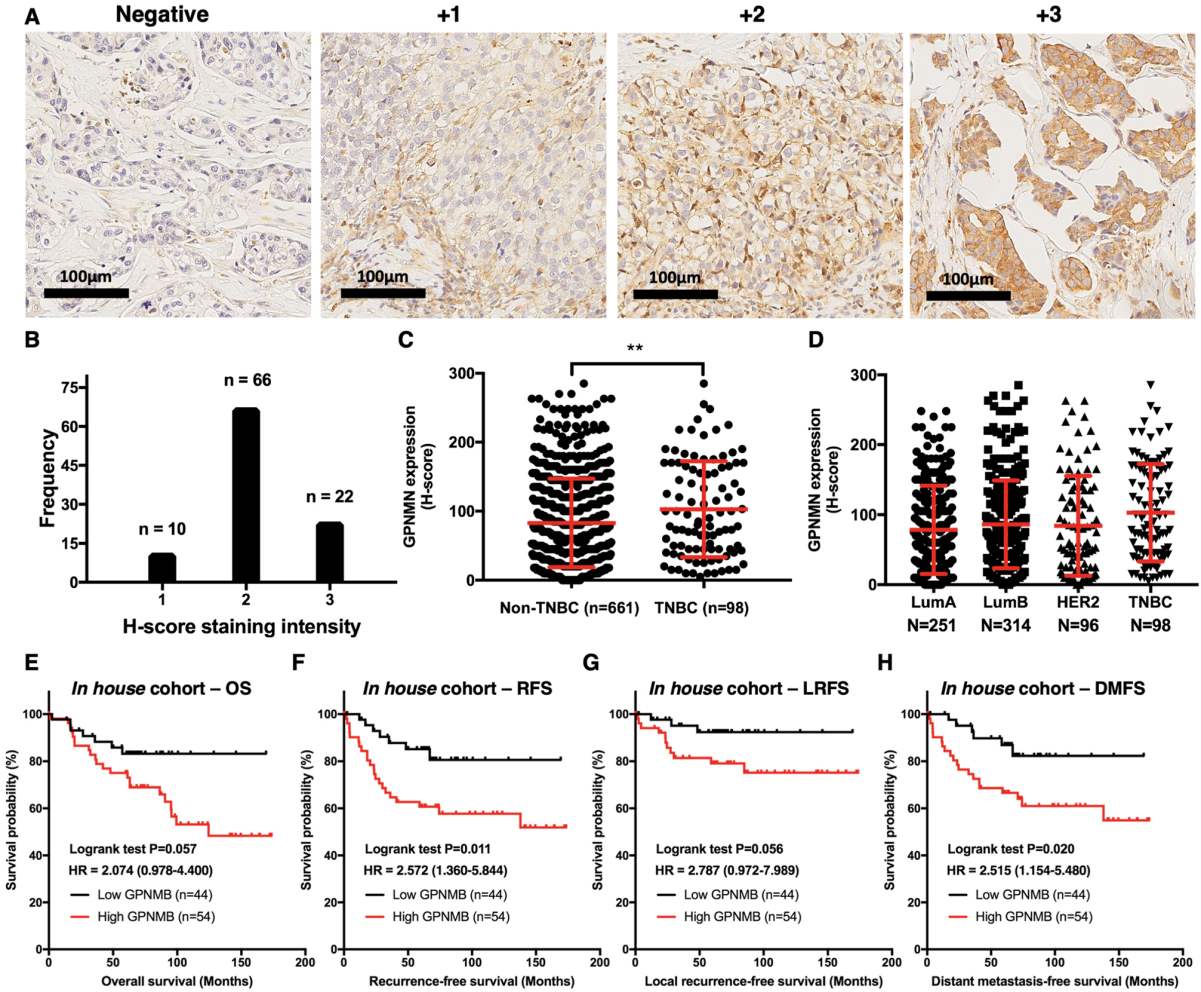
GPNMB expression in patients with triple-negative breast cancer (TNBC) is a predictor of breast cancer recurrence. (**A**) Representative immunohistochemistry pattern of GPNMB intensity graded as 0, negative-staining; 1 + , weak; 2 + , moderate and 3 + , strong. (**B**) Immunohistochemistry showing the GPNMB staining intensity among patients with TNBC. (**C**) Quantitative expression of GPNMB was compared in TNBC and non-TNBC groups. T test, **P < 0.01. (**D**) Quantitative expression of GPNMB was compared in immunohistochemical subtypes. (**E–H**) Kaplan–Meier analysis of the influence of GPNMB protein expression on (**E**) Overall survival, OS, *P* = 0.057. (**F**) Recurrence-free survival, RFS, *P* = 0.011. (**G**) Local recurrence-free survival, LRFS, *P* = 0.056. (**H**) Distant metastasis-free survival, DMFS, *P* = 0.020.

To investigate the clinical significance of GPNMB in primary breast cancer, we quantified GPNMB expression of each specimen using IHC. The representative IHC stainings of GPNMB in the breast tissue were illustrated (Fig. 1A). The intensity of GPNMB expression in TNBC sample was analyzed which showed mostly moderate staining (Fig. 1B). Furthermore, we calculated the percentage of positive-stained carcinoma cells and analyzed the H-score between subgroups. In TNBC, the mean and medium of GPNMB expression were 102.9 and 87.5 with interquartile range from 45.0 to 165.0. The average H-scores of GPNMB in TNBC subtype were significantly higher than those in non-TNBC subtype (102.9 vs. 82.9, *P* = 0.0045; Fig. 1C). The average H-scores of GPNMB in luminal A, luminal B, HER2 and TNBC were 78.4, 86.2, 84.0 and 102.9 respectively (Fig. 1D). These results demonstrated that GPNMB might be overexpressed preferentially in TNBC.

### GPNMB expression in TNBC is associated with worse recurrence-free and distant metastasis-free survival

To determine the clinical relevance of this observation, we classified the patients with TNBC into high- and low-expression GPNMB subgroups. The clinical and pathological characteristics were analyzed which showed no statistically significant correlation in characteristics including age, T value, N value, stage or the presence of lymphovascular invasion (Table 2). In terms of histologic grade, we found a correlation between GPNMB overexpression and histologic grade (*P* = 0.001). This indicated GPNMB expression was enriched in low histologic grade TNBC tumor.

**Table 2:** Association of clinical and pathological characteristics with GPNMB expression in 98 patients with TNBC.

Characteristics	Number of patients (n = 98)	GPNMB expression		*P*-value
		Low (n = 44)	High (n = 54)	
**Age**				
≤ 60	60	24 (54.5)	36 (66.7)	0.221
> 60	38	20 (45.5)	18 (33.3)	
**Tumor stage**				
Tis	1	0 (0)	1 (1.9)	0.530
T1	31	13 (29.5)	18 (33.3)	
T2	59	28 (63.6)	31 (57.4)	
T3	2	0 (0)	2 (3.7)	
T4	5	3 (6.8)	2 (3.7)	
**Nodal status**				
N0	56	27 (61.4)	29 (53.7)	0.558
N1	22	8 (18.2)	14 (25.9)	
N2	7	2 (4.5)	5 (9.3)	
N3	13	7 (15.9)	6 (11.1)	
**TNM stage**				
DCIS + I + II	75	33 (75.0)	42 (77.8)	0.813
III	23	11 (25.0)	11 (22.2)	
Grade				
1 + 2	36	8 (18.2)	28 (51.9)	0.001
3	62	36 (81.8)	26 (48.1)	
**Lymphovascular invasion**				
Absent	76	31 (72.1)	45 (83.3)	0.182
Present	21	12 (27.9)	9 (16.7)	
NA^a^	1			
**Tumor necrosis**				
Absent	41	20 (45.5)	21 (38.9)	0.512
Present	57	24 (54.5)	33 (61.1)	

^a^NA, not available.

To evaluate the prognostic value of GPNMB expression in patients with TNBC, we analyzed the effects of GPNMB overexpression in Kaplan–Meier survival curve (Fig. 1E–H). After a medium follow-up of 74 months, the OS, RFS, LRFS (local recurrence-free survival) and DMFS for patients with TNBC were 70.4%, 70.4%, 85.7% and 73.5% respectively. There were statistically significant correlations in RFS (*P* = 0.011, HR = 2.572, CI  1.360–5.844; Fig. 1F) and DMFS (*P* = 0.020, HR = 2.515, CI  1.154–5.480; Fig. 1H). In contrast, survival curve in OS and LRFS showed only trend but no statistically significant correlation (Fig. 1E,G).

Furthermore, multivariate analysis by Cox regression showed GPNMB overexpression was an unfavorable independent factor associated with RFS and DMFS (*P* = 0.008, HR = 3.22, CI  1.36–7.61; *P* = 0.017, HR = 3.08, CI  1.22–7.74, Table 3). The presence of lymphovascular invasion did not showed prognostic outcomes in RFS and DMFS multivariate analysis (*P* = 0.136, HR = 2.16, CI  0.78–5.97; *P* = 0.259, HR = 1.88, CI  0.62–5.67). As expected, TNM stage according to the American Joint Committee on Cancer demonstrated statistically significant in predicting RFS and DMFS (*P* < 0.001, HR = 4.58, CI  2.19–9.56; *P* < 0.001, HR = 4.02, CI  1.85–8.75).

**Table 3:** Univariate and multivariate Cox analysis of factors associated with recurrence free survival (RFS) and distant metastasis-free survival (DMFS) in patients with TNBC.

	Univariate RFS analysis		Multivariate RFS analysis	
	Hazard ratio (95% CI)	*P*	Hazard ratio (95%CI)	*P*
GPNMB H-score (> 75.5 vs. ≤ 75.5)	2.57 (1.36–5.84)	0.011	3.22 (1.36–7.61)	0.008
Lymphovascular invasion (present vs. absent)	3.45 (1.65–7.19)	< 0.001	2.16 (0.78–5.97)	0.136
Grade (3 vs. 2–1)	0.56 (0.27–1.17)	0.119	–	–
Stage (III vs. II–I–DCIS)	4.13 (1.99–8.59)	< 0.001	4.58 (2.19–9.56)	< 0.001

	Univariate DMFS analysis		Multivariate DMFS analysis	
	Hazard ratio (95% CI)	*P*	Hazard ratio (95%CI)	*P*
GPNMB H-score (> 75.5 vs. ≤ 75.5)	2.51 (1.15–5.48)	0.020	3.08 (1.22–7.74)	0.017
Lymphovascular invasion (present vs. absent)	2.52 (1.26–5.02)	0.006	1.88 (0.62–5.67)	0.259
Grade (3 vs. 2–1)	0.64 (0.32–1.24)	0.183	–	–
Stage (III vs. II–I–DCIS)	3.72 (1.71–8.07)	0.001	4.02 (1.85–8.75)	< 0.001

Apart from 98 patients with TNBC, we analyzed total 759 patients with primary breast cancer and showed GPNMB overexpression had no prognostic effect in RFS (*P* = 0.118; Additional file 1: Fig. S1B), LRFS (*P* = 0.432; Additional file 1: Fig. S1C) and DMFS (*P* = 0.182; Additional file 1: Fig. S1D) but OS (*P* = 0.016; Additional file 1: Fig. S1A). It corresponded with our previous results that GPNMB correlated with worse outcome in TNBC subtype but not in all breast cancer subtypes. Overall, these results implicated GPNMB overexpression is a biomarker of poor prognosis in TNBC and may lead to breast cancer recurrence with distant metastasis.

### GPNMB overexpression might lead to poor survival outcome

**Figure 2:**
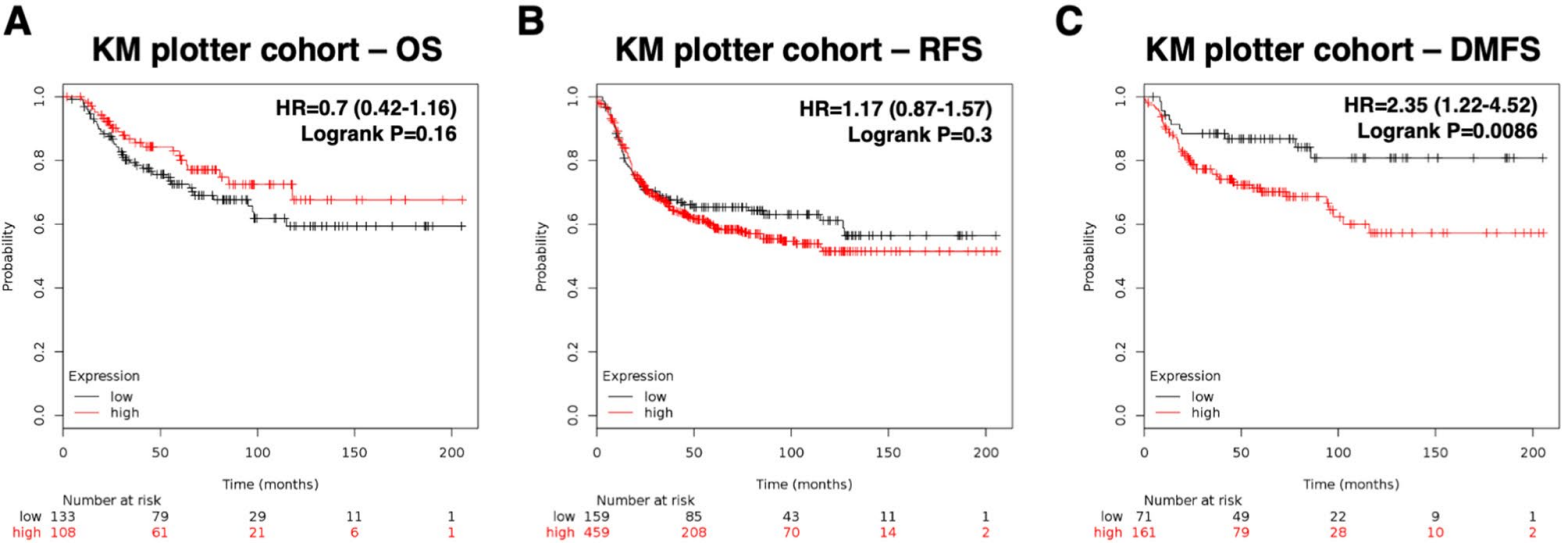
Overexpression of GPNMB mRNA correlates with worse DMFS. Kaplan–Meier analysis of the influence of GPNMB mRNA expression on (**A**) OS, *P* = 0.16. (**B**) RFS, *P* = 0.3. (**C**) DMFS, *P* < 0.01 in patients with TNBC from the public database (http://kmplot.com).

To further examine the significance of GPNMB overexpression in TNBC, we next evaluated its expression in mRNA level in silico. Among the cohort of 5143 patients with breast cancer from public data KM plotter^30^, 879 (17.0%) patients were classified as basal subtype breast cancer. Among these TNBC patients, we analyzed the GPNMB overexpression effect in Kaplan–Meier survival curve. As a result, no statistically significant correlation was found in OS (*P* = 0.16; Fig. 2A) and RFS (*P* = 0.3; Fig. 2B). Nevertheless, unfavorable prognostic factor was found in DMFS analysis (*P* < 0.01, HR = 2.35, CI  1.22–4.52; Fig. 2C). These outcomes were in consistence with our in-house data analysis which showed GPNMB mRNA overexpression in TNBC may promote breast cancer recurrence especially in terms of distant metastasis.

### GPNMB expression is positively associated with EMT pathways

**Figure 3:**
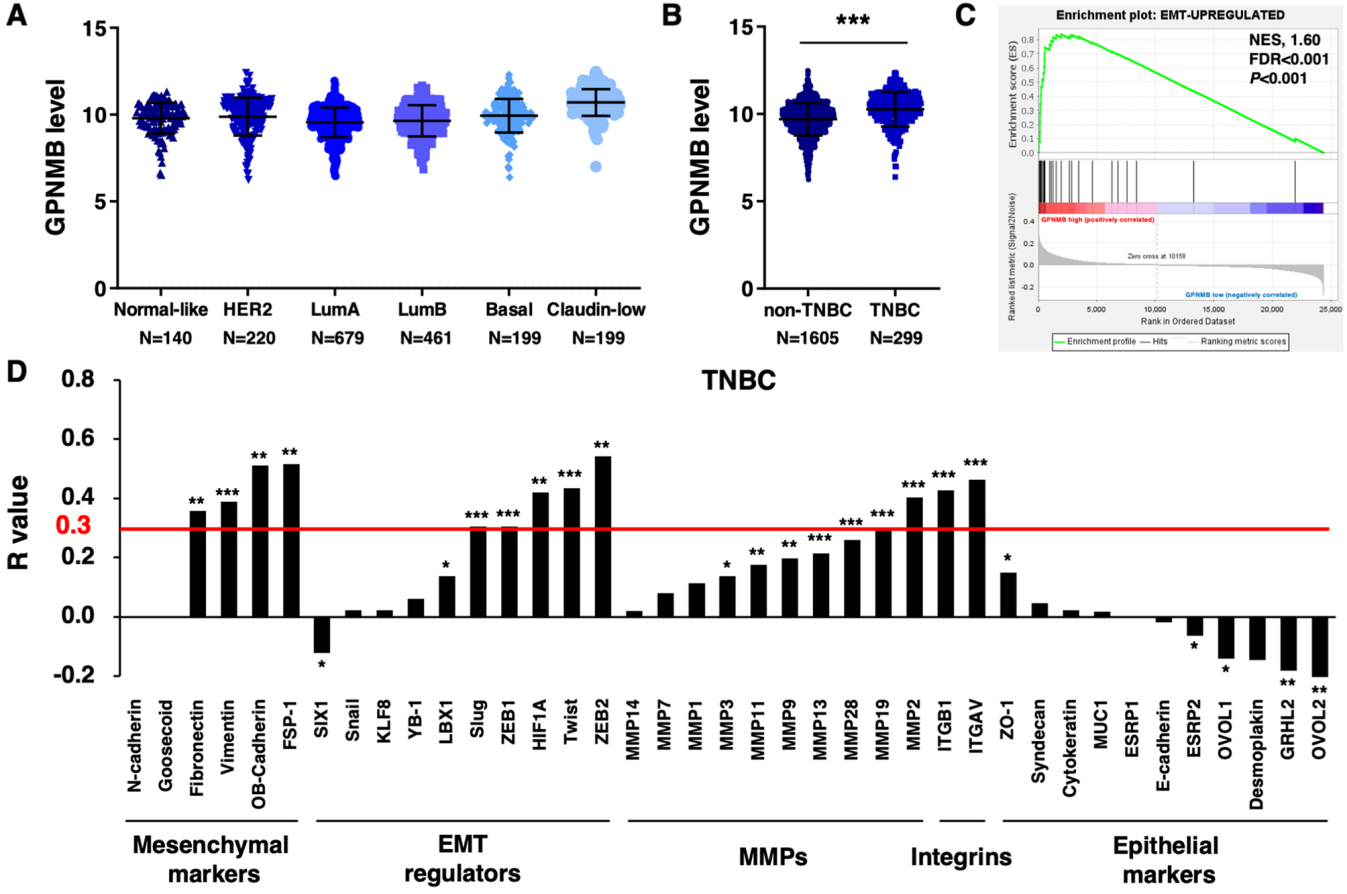
GPNMB is overexpressed in TNBC patients and correlated with EMT-associated genes. (**A**–**D**) GPNMB transcript data from Molecular Taxonomy of Breast Cancer International Consortium (METABRIC) in cBioPortal was analyzed. (**A**) Expression of GPNMB transcript was compared based on PAM50 classification. (**B**) TNBC harbored higher GPNMB level compared to non-TNBC based on gene expression classification. ***P < 0.001. (**C**) Gene set enrichment analysis (GSEA) of METABRIC data showed enrichment of an EMT-upregulated signature in GPNMB high patients. (**D**) The correlations of GPNMB with mesenchymal markers, EMT regulators, MMPs genes, integrins and epithelial markers were analyzed. The Pearson correlation coefficient above 0.3 was considered as positive correlation.

Since GPNMB was shown to promote recurrence in TNBC, we next aimed to investigate the possible mechanism of how GPNMB could affect the behavior in TNBC. We analyzed a collection of 2000 breast tumors in the data set of METABRIC from cBioPortal^31,32^. We found that basal and claudin-low subtypes had GPNMB overexpression in contrast to other breast cancer subtypes based on gene expression classifier PAM50 (Fig. 3A). Furthermore, GPNMB level was statistically significant higher in TNBC than non-TNBC based on expression-based classification (*P* < 0.001; Fig. 3B). GPNMB expression evaluated from CCLE cohort data set also revealed higher GPNMB level in basal and claudin-low subtype breast cancer cell lines (Additional file 1: Fig. S2A)^33^. Of importance, we found a significant enrichment of EMT-upregulated genes in GPNMB high expression TNBC patients (normalized enrichment score, NES, 1.60; FDR < 0.001; *P* < 0.001; Fig. 3C). We further explored the correlation in invasion and metastasis gene markers and ranked the Pearson correlation coefficients in ascending order. The results revealed GPNMB transcripts levels were positively correlated with transcript levels of mesenchymal markers, EMT regulators, pro-invasive MMPs and integrins (Fig. 3D). The Pearson correlation coefficient above 0.3 was considered as positive correlation^34^. Accordingly, we performed 4 extra IHC markers for EMT and found E-cadherin was significantly reversely associated with GPNMB, while other markers seemed non-significant (Additional file 1: Fig. S2B and Table S1). We found some representative cases with typically reciprocal expressions of the four markers (Additional file 1: Fig. S2C). In general, E-cadherin are negatively associated with GPNMB, whereas the correlations of GPNMB with other epithelial or mesenchymal markers seemed not statistically significant. Despite the statistical non-significance, epithelial ZO-1 showed a trend toward negative correlation with GPNMB, whereas mesenchymal markers showed a trend toward positive correlation with GPNMB (Table S1).

### GPNMB enhances cell invasion via MMP pathway

**Figure 4:**
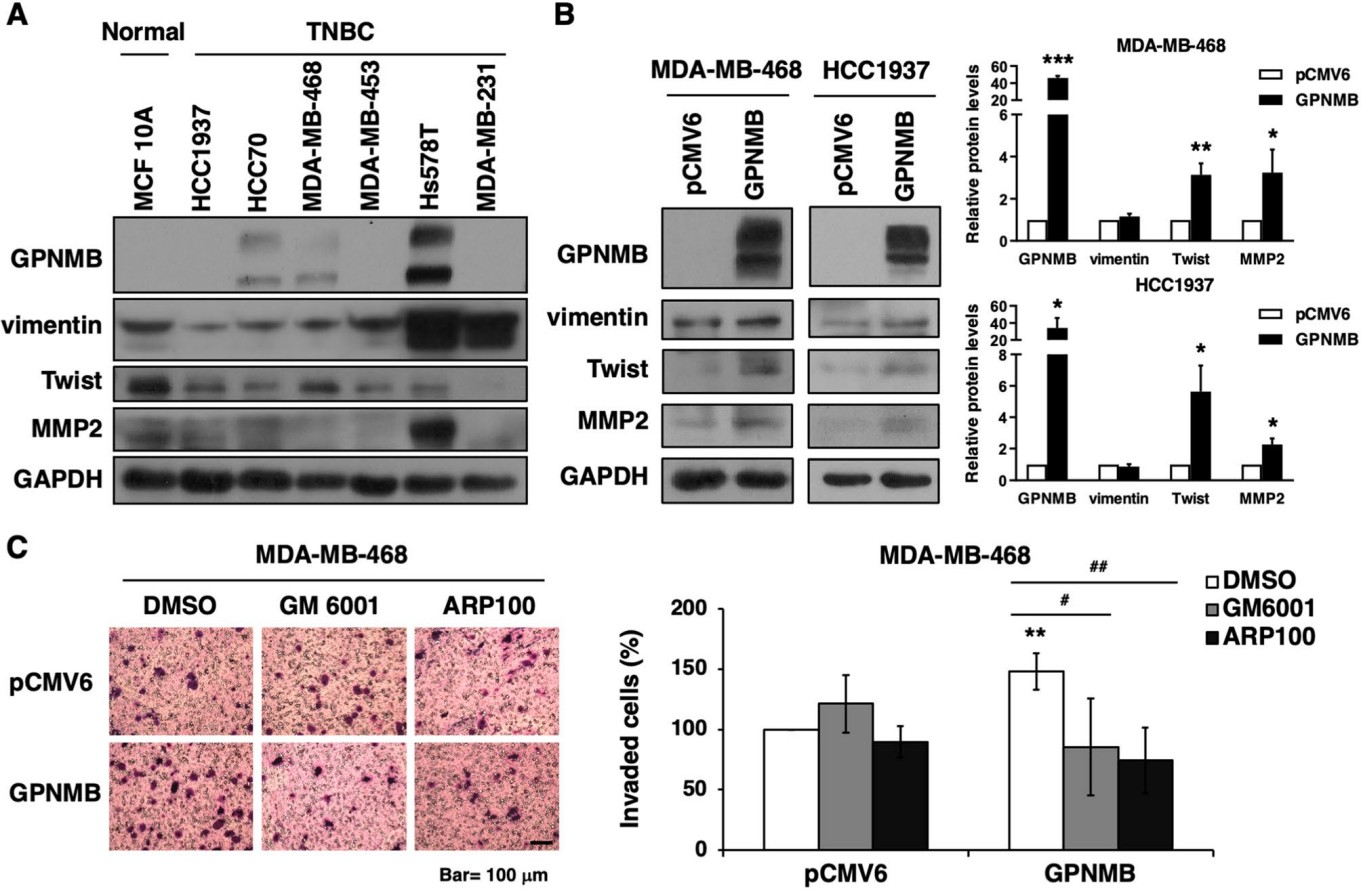
GPNMB drives cell invasion through MMP2. (**A**) Whole-cell extracts of MCF 10A epithelial cells and TNBC cell lines were examined by Western blot analysis. (**B**) MDA-MB-468 and HCC1937 cells transfected with GPNMB expression construct or control plasmids (pCMV6) for 48 h were analyzed by Western blot analysis (left). The Western blottings were quantified (right). (**C**) MDA-MB-468 cells were transfected with GPNMB-expressing or pCMV6 plasmids for 48 h. The transfected cells were resuspended in DMEM serum free medium containing DMSO, GM 6001 (20 μM) or ARP100 (20 μM) for invasion assay. Data are representative of three independent experiments. *P < 0.05, **P < 0.01, ***P < 0.001, #*P* < 0.05, ##*P* < 0.01.

Then, we further validate the association between GPNMB and cell invasion in vitro. Results of Western blot analysis showed that TNBC cell lines harbored variable levels of GPNMB, vimentin, Twist and MMP2. Three TNBC cell lines had strong GPNMB expression compared to MCF 10A breast epithelial cell lines (Fig. 4A). Overexpression of GPNMB in MDA-MB-468 and HCC1937 cells increased the protein levels of Twist and MMP2 but not vimentin (Fig. 4B). To evaluate whether GPNMB regulated cell invasion through MMP2, the broad spectrum MMP inhibitor (GM 6001) and the selective inhibitor of MMP-2 (ARP100) were used for in vitro study. Data exhibited GPNMB-drove cell invasion was suppressed by GM 6001 and ARP100 (Fig. 4C). These findings suggested that GPNMB might affect the EMT and MMP pathways and lead to metastasis.

## Discussion

In the current study, we demonstrated the clinical significance of GPNMB expression in patients with TNBC. The statistical analysis indicates that the transmembrane glycoprotein GPNMB was overexpressed in TNBC at both protein and mRNA level (Figs. 1C,D, 3A,B). We also discovered that overexpression of GPNMB lead to poor clinical outcome especially distant metastasis in patients with TNBC (Figs. 1F,H, 2C). These results led us to explore the possible mechanism of how GPNMB promotes distant metastasis. We searched the METABRIC database in silico and discovered the correlations between GPNMB and EMT regulators as well as pro-invasive MMPS and integrin. The protein levels of Twist and MMP2 were upregulated by GPNMB overexpression. GPNMB-enhanced cell invasion was attenuated by MMP inhibitors (Fig. 4). These results implicated that GPNMB may play an important role in the distant recurrence, offering a prognostic biomarker and potentially therapeutic targets in patients with TNBC.

Transmembrane protein GPNMB has been reported overexpressed preferentially in many types of cancer tissues relative to normal tissues^15–22^. Kuan et al. demonstrated correlations of GPNMB protein and mRNA expression by Spearman’s analysis^22^. Our *in-house* data was explored at protein level by IHC and further confirmed by in silico data at mRNA level. As expected, both protein and mRNA GPNMB expressions correlated with poor clinical survival and acted as a prognostic predictor in TNBC. In previous study, GPNMB mRNA expression was shown highly enriched in basal-like subtypes and correlated with recurrence in patients with TNBC^5^. Our findings supported the preferential expression of GPNMB in TNBC and discovered the higher rate of recurrence, especially distant recurrence in patients with GPNMB overexpression.

Distant metastasis is the major cause of cancer mortality in patients with breast cancer and more than half of the first presentation of metastatic breast cancer is bone^35^. GPNMB, also known as osteoactivin, has been identified as a protein expressed in aggressive breast cancer and promoted breast cancer metastasis to bone^15^. James et al. demonstrated that bone metastasis was significantly more often associated with well differentiated primary breast cancer than high grade tumor^36^. In our study, we found high GPNMB level is correlated with low histologic grade or well differentiated tumor, whereas depletion of GPNMB lead to high histologic grade or less differentiated tumor.

The mechanism of tumor recurrence and the exact role of GPNMB in breast cancer is still under discussion. In vitro study of GPNMB-expressing tumor cells showed no impact on cellular growth or death but increased expression of MMP-3 and MMP-9 as well as increased invasiveness^15,26,37^. Maric et al. demonstrated GPNMB enhanced breast cancer cell adhesion to fibronectin and formed a complex with integrin α5β1 expressed on the cells, such as endothelial cells to activate downstream pathways^29,38^. Breast cancer cells expressed high level of cell surface-GPNMB showed elevated cancer stem cells genes and EMT transcriptional factors genes exhibiting high tumorigenicity^39^. In addition, phosphorylation of GPNMB was involved in glycolysis reprogramming of TNBC and associated with worse outcome in TNBC patients^40^. Nonetheless, breast cancer is a heterogenous disease and different subtypes may influence the mechanism and signal pathway underpinning the GPNMB^41,42^. In our study, we focused on TNBC specimens and analyzed the GPNMB expression by Pearson correlation with data set from METABRIC database. We presented the correlation between GPNMB mRNA expression and EMT regulators expression. Several EMT regulators and mesenchymal marker vimentin as well as MMPs and integrin gene were associated with GPNMB overexpression in TNBC. Notably, our data exhibited the dissimilar data between transcription and translational levels (Fig. 3 and S2). It is possible that other EMT-regulatory pathways may interfere with the expression associations between GPNMB and these markers, more studies are necessary. Nevertheless, our study and others have shown that GPNMB indeed is linked to EMT pathways^43^, and other yet-to-be identified regulatory mechanisms may contribute to the discrepancy for the association findings from public database (METABRIC) and IHC-based tumor sample studies. We also noticed although the expression of vimentin was low in GPNMB low expression tumor part, the expression of vimentin was high in its stroma compartment, which may act as a measure of internal quality control in IHC^44,45^. Our data also revealed that GPNMB increased Twist and MMP2 expressions in vitro*.* Overexpression of GPNMB promoted TNBC cell invasion which was declined by MMP inhibitors*.* Altogether, these findings implicated that GPNMB overexpression in patients with TNBC may have higher chances of distant metastasis by affecting EMT pathway.

In our study, Kaplan–Meier survival curve showed a consistency in poor DMFS outcomes in both mRNA and protein level. However, there are discrepancies between mRNA and protein level in OS and RFS. There have been many studies on the differential expression between mRNA and protein level in yeasts and human tissue, and the results have been relatively inconsistent^46,47^. There are several possible explanations for why mRNA levels of the tested genes are inconsistent with its corresponding protein levels. For example, the difference in protein degradation and translation as well as some messengers are transcribed but not translated would influence the number of mRNA copies^48^. Consequently, the number of mRNA copies may not necessarily reflect the number of functional protein molecules. Moreover, the discrepancies in regulatory mechanisms of different genes, mRNA or proteins among different cell types or tumor heterogeneity may also contribute to the inconsistency of mRNA and protein levels of certain genes. Therefore, we suggest that mRNA expression might not be perfect in predicting protein expression levels. Nevertheless, they should provide us with a more detailed and comprehensive understanding of gene function overall.

Currently ADCs targeting GPNMB-expressing cancers are under investigation, although Glembatumumab vedotin failed to demonstrated clinical advantages over capecitabine on progression-free survival in the phase IIb METRIC study (NCT1997333), several interesting points remained to be discussed. GPNMB had been identified as an oncogene in TNBC and GPNMB-expressing cancer cells could be killed by a GPNMB-targeting ADC^5^. The tumor-killing effect of GPNMB-targeting ADC was based on low expression of surface GPNMB in normal cells but high expression of GPNMB in cancer cells. Our study provided that the majority of TNBC specimens were stained moderate intensity (2 +) in GPNMB IHC, suggesting a hypothesis that selecting stronger intensity (such as 3 +) GPNMB-expressing TNBC patients might be more suitable candidates for GPNMB-targeting ADC. Furthermore, combination therapy that coupling other treatment modality such as sheddase inhibitors or tyrosine kinase inhibitors might enhance the efficacy of GPNMB-targeting ADC^29^. These warranted further investigations into the potential of enhancing the benefits of GPNMB-targeting ADC.

There are limitations to this study. First, within these 759 patients with a medium follow-up of 74 months, bias can be drawn from changes in different clinical treatment course of patients. Furthermore, studies including knockout of GPNMB in vitro or xenograft assays are needed to assess the biological role of GPNMB, although prior studies have demonstrated GPNMB-targeting ADC could inhibit growth of tumor and induce complete regression in melanoma xenografts^49,50^. Knockdown of GPNMB could reduce melanoma growth in immunocompetent mice^51^. GPNMB overexpression in metastatic mouse mammary tumor cells could upregulate tumor growth, stimulate bone metastasis and induce EMT^15^. These previous studies suggested that GPNMB might be a potential biomarker in cancer biology. Further evidences are needed to conclude the mechanism behind GPNMB promoting distant metastasis.

Our study demonstrated that GPNMB was overexpressed in TNBC at both protein and gene levels. GPNMB overexpression in patients with TNBC correlates with poor clinical outcomes in terms of recurrence and distant metastasis. Furthermore, GPNMB may be associated with distant metastasis, at least in part, through cross talk with EMT pathway. Our study provided insights into the important role of GPNMB in TNBC and might be a surrogate marker for predicting the clinical outcomes.

## Methods

### Patients

We enrolled 759 patients diagnosed with breast cancer who underwent primary surgery between 2001 and 2010 from Taipei Veterans General Hospital (Taipei, Taiwan). The last follow up cut-off was February 24, 2016. Clinico-pathological characteristics including TNM staging, histological grade, and tumor type were determined in accordance with the WHO classification system. The clinical parameters were collected from medical records by oncologists. Representative areas of each tumor were carefully selected and constructed into tissue microarrays (TMA) as previously described^52^. This study was approved by the ethics committee of the Institutional Review Board of Taipei Veterans General Hospital. Informed consent was obtained and conducted in compliance with the Helsinki Declaration.

### Determination of histology score (H-score)

H-score was calculated by using a semi-quantitative assessment of the percentage of positive-stained carcinoma cells (from 0 to 100) and the staining intensity (graded as 0, non-staining; 1, weak; 2, moderate and 3, strong). The range of H-score was from 0 to 300. Board certified pathologists specialized in breast cancer evaluated and scored the H-score independently without given the clinical information of tumor specimen, as previously described^53^. Expression level of GPNMB was categorized into high and low subgroup according to the cut-off value determined by ROC curve analysis.

### Immunohistochemistry

Immunostainings were performed on 4 μm paraffin-embedded tissue sections. The sections were deparaffinized and rinsed with 10 mM Tris–HCl (pH 7.4) and 150 mM sodium chloride. Peroxidase was quenched with methanol and 3% hydrogen peroxide. Slides were placed in 10 mM citrate buffer (pH 6.0) at 100 °C for 20 min in a pressurized heating chamber^53^. Tissue sections were incubated with the antibodies against GPNMB (1:200; AI12434, Abgent), E-cadherin (1:200; Clone GM016, Genemed), ZO-1 (1:200; 61–7300, Invitrogen), N-cadherin (1:20; NCL-L-N-Cad, Leica Biosystems) and vimentin (1:200; clone V9, Leica Biosystems) for 1 h at room temperature and then washed with phosphor-buffered saline. Bound antibodies were detected using the EnVision Detection Systems Peroxidase/DAB, Rabbit/Mouse kit (Dako, Denmark). The slides were counterstained with hematoxylin. For negative controls, above IHC steps were performed without primary antibody. Mouse monoclonal antibodies were used to detected ER (M7047, Dako), PR (M3569, Dako), and erbB-2 (28-0003z, Invitrogen). Tissues were identified as ER or PR positive breast cancer if more than 10% nuclei were stained and as Her2-positive breast cancer if 3 + Her2 expression was scored.

### In silico survival analysis with public open databases

Kaplan–Meier survival analysis of patients with basal intrinsic subtype breast cancer was done based on mRNA expression of GPNMB (Affymetrix probe ID 201,141 _at) and survival endpoint including overall survival (OS), recurrence-free survival (RFS) and distant metastasis-free survival (DMFS) from KM plotter (https://kmplot.com) ^30^. An auto-selected optimal cut-off was selected for analysis. About 2000 breast cancer GPNMB gene expression data from five different research centers in the UK and Canada was evaluated from Molecular Taxonomy of Breast Cancer International Consortium (METABRIC) in cBioPortal (http://www.cbioportal.org/)^31,32^. Supplemental validation data of correlation between GPNMB gene expression and breast cancer cell lines subtypes was analyzed from Cancer Cell Line Encyclopedia (http://www.broadinstitute.org/ccle; CCLE)^33^. To assess gene sets were enriched in GPNMB high patients Gene Set Enrichment Analysis (GSEA) (http://software.broadinstitute.org/gsea/index.jsp) was used^54^. A *P*-value < 0.05 and false discovery ratio (FDR) < 0.25 were considered as statistical significance.

### Cell culture, reagents and transfection

MCF10A epithelial cell line and breast cancer cell lines were purchased from American Type Culture Collection (Manassas, VA). All cells were maintained in Dulbecco's Modified Eagle Medium (DMEM) or RPMI-1640 Medium (Gibco) supplement with 10% FBS, 2-mM L-glutamine, 0.1-mM non-essential amino acids and 1% penicillin/streptomycin in a 5% CO_2_ atmosphere at 37 °C. GM 6001 and ARP100 purchased from Santa Cruz Biotechnology (Dallas, TX, USA) were dissolved in dimethyl sulfoxide (DMSO). The GPNMB and pCMV6 expression constructs were purchased from OriGene (Rockville, MD, USA). For transfection, cells were seeded into 6-cm dish for 24 h and transfected using Lipofectamine 3000 (Thermo Fisher Scientific, Waltham, MA, USA) following the manufacturer’s instructions.

### Western blot analysis

The whole-cell extracts were lysed in RIPA buffer with a Halt Protease and Phosphatase Inhibitor Cocktail (Thermo Fisher Scientific). The Laemmli’s sample buffer was added to the lysates and boiled at 95 °C for 5 min then analyzed by sodium dodecyl sulfate–polyacrylamide gel electrophoresis. Western blotting was performed with anti-GPNMB, anti-vimentin, anti-Twist, anti-MMP2 and anti-GAPDH antibodies (Cell Signaling Technology, Danvers, MA, USA). Quantification of protein levels was analyzed by ImageJ software.

### Invasion assay

The invasion assay was performed in 24-well plate for 20 h. The transfected cells (3 × 10^4^) in 200 μL of serum free medium were seeded onto upper Matrigel matrix (Corning, New York, NY, USA) coated Cell Culture Insert (Greiner Bio One, Kremsmünster, Austria). The lower chamber contained 900 μL of complete culture medium. The invaded cells were fixed with methanol for 10 min and stained with 0.005% crystal violet for 1 h at room temperature. The numbers of invaded cells were counted under the microscope from 10 random fields^55^.

### Statistical analysis

OS was defined as the time from surgery until death. RFS was defined as the time from surgery until any primary or distant recurrence with an appearance of secondary tumor or death. LRFS was defined as the time from surgery until any partial response, progressive disease, local recurrence or death. DMFS was defined as the time from surgery until any distant recurrence of cancer or death. Statistical analysis was performed with SPSS 24.0 software (SPSS, Chicago, IL, USA). Using ROC curve, the optimal cut-off value of H-scores was determined. Survival curves were performed by Kaplan–Meier method and compared with log-rank test. Data analysis was performed using Student’s t-test^53^. A *P*-value less than 0.05 was considered statistically significant.

### Ethics approval

All procedures performed in studies involving human participants were in accordance with the ethical standards of the institutional and national research committee and with the 1964 Helsinki declaration and its later amendments or comparable ethical standards.

### Informed consent

The ethics committee of the Institutional Review Board of Taipei Veterans General Hospital approved this study. Tumors for immunohistochemical study were collected in accordance with the Declaration of Helsinki and informed consents from sample donors were obtained at time of their donation.

## Supplementary Information

Supplementary Information.

## Data Availability

The data that support the gene expression is evaluated from the database Kaplan–Meier Plotter (http://kmplot.com), Molecular Taxonomy of Breast Cancer International Consortium (METABRIC) in cBioPortal (http://www.cbioportal.org/) and Cancer Cell Line Encyclopedia (http://www.broadinstitute.org/ccle, CCLE). Other data analyzed in the current study is available from the corresponding author on reasonable request.

## Abbreviations

GPNMB: Glycoprotein non-metastatic B
TNBC: Triple-negative breast cancer
ADC: Antibody–drug conjugate
IHC: Immunohistochemistry
DMFS: Distant metastasis-free survival
RFS: Recurrence-free survival
OS: Overall survival
LRFS: Local recurrence-free survival
EMT: Epithelial-mesenchymal transition
MMP: Matrix metalloprotease
HGFIN: Hematopoietic growth factor inducible neurokinin-1
DC-HIL: Dendritic cell-associated heparin sulfate proteoglycan-dependent integrin ligand
METABRIC: Molecular Taxonomy of Breast Cancer International Consortium
CCLE: Cancer Cell Line Encyclopedia
