# Supplementary material for: Expression pattern and prognostic impact of glycoprotein non-metastatic B (GPNMB) in triple-negative breast cancer
Source: Sci Rep. 2021 Jun 9;11:12171. doi: 10.1038/s41598-021-91588-3 (PMC8190094; doi:10.1038/s41598-021-91588-3)

Expression pattern and Prognostic impact of Glycoprotein non-metastatic B (GPNMB) in Triple-Negative Breast Cancer

Yu-Hsiang Huang[1†], Pei-Yi Chu[2, 3†], Ji-Lin Chen[4,5], Chun-Teng Huang[1,6], Chi-Cheng Huang[4,7,8], Yi-Fang Tsai[1,4,7], Yu-Ling Wang[4,9], Pei-Ju Lien[4,10], Ling-Ming Tseng[1,4,9], Chun-Yu Liu[1,4,5,11*]

[1]School of Medicine, National Yang Ming Chiao Tung University, Hsinchu, Taiwan;
[2]Department of Pathology, Show Chwan Memorial Hospital, Changhua City, Taiwan;
[3]School of Medicine, Fu Jen Catholic University, New Taipei City, Taiwan;
[4]Comprehensive Breast Health Center, Taipei Veterans General Hospital, Taipei, Taiwan;
[5]Division of Medical Oncology, Department of Oncology, Taipei Veterans General Hospital, Taipei, Taiwan;
[6]Division of Hematology & Oncology, Department of Medicine, Yang-Ming Branch of Taipei City Hospital, Taipei, Taiwan;
[7]Division of General Surgery, Department of Surgery, Taipei Veterans General Hospital, Taipei, Taiwan
[8]Department of Public Health, College of Public Health, National Taiwan University, Taipei, Taiwan
[9]Division of Experimental Surgery, Department of Surgery, Taipei Veterans General Hospital, Taipei, Taiwan;
[10]Department of Nursing, Taipei Veterans General Hospital, Taipei, Taiwan;
[11]Division of Transfusion Medicine, Department of Medicine, Taipei Veterans General Hospital, Taipei, Taiwan;

[†]Yu-Hsiang Huang and Pei-Yi Chu contributed equally to the work.

Correspondence to:

Chun-Yu Liu, MD, PhD
Division of Transfusion Medicine, Department of Medicine, Taipei Veterans General Hospital. No. 201, Sec. 2, Shih-Pai Road, Taipei 112, Taiwan.
Tel: 886-2-28712121 ext. 7529
Fax: 886–2-28757762
E-mail: cyliu3@vghtpe.gov.tw

Table S1. Relationship of GPNMB expression with expressions of EMT markers

Characteristics	GPNMB expression		*P* value
	Low	High	
E-cadherin			
High	15 (51.7)	5 (18.5)	0.002
Medium	9 (31.0)	8 (29.6)	
Low	3 (10.3)	14 (51.9)	
NA	2 (6.9)	0 (0.0)	
ZO-1			
High	18 (62.1)	10 (37.0)	0.073
Medium	3 (10.3)	9 (33.3)	
Low	8 (27.6)	8 (29.6)	
N-cadherin			
High	13 (44.8)	9 (33.3)	0.519
Medium	6 (20.7)	9 (33.3)	
Low	10 (34.5)	9 (33.3)	
vimentin			
High	11 (37.9)	8 (29.6)	0.112
Medium	7 (24.1)	13 (48.1)	
Low	11 (37.9)	5 (18.5)	
NA	0 (0.0)	1 (3.7)	

The cutoff value of the H-score of Trop-2 was selected as 138.75.
NA, not available.

Figure S1

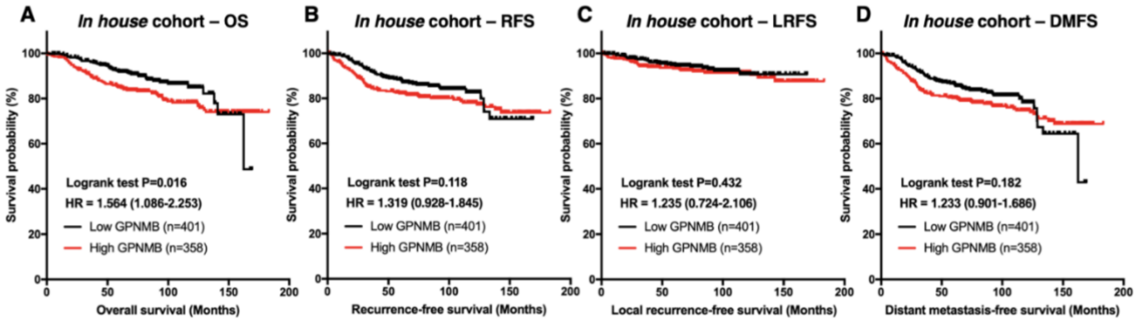

Figure S1. GPNMB is a prognostic biomarker in overall survival in patients with breast cancer. Kaplan-Meier analysis of the influence of GPNMB IHC expression on (A) OS (B) RFS (C) LRFS (D) DMFS in patients with breast cancer.

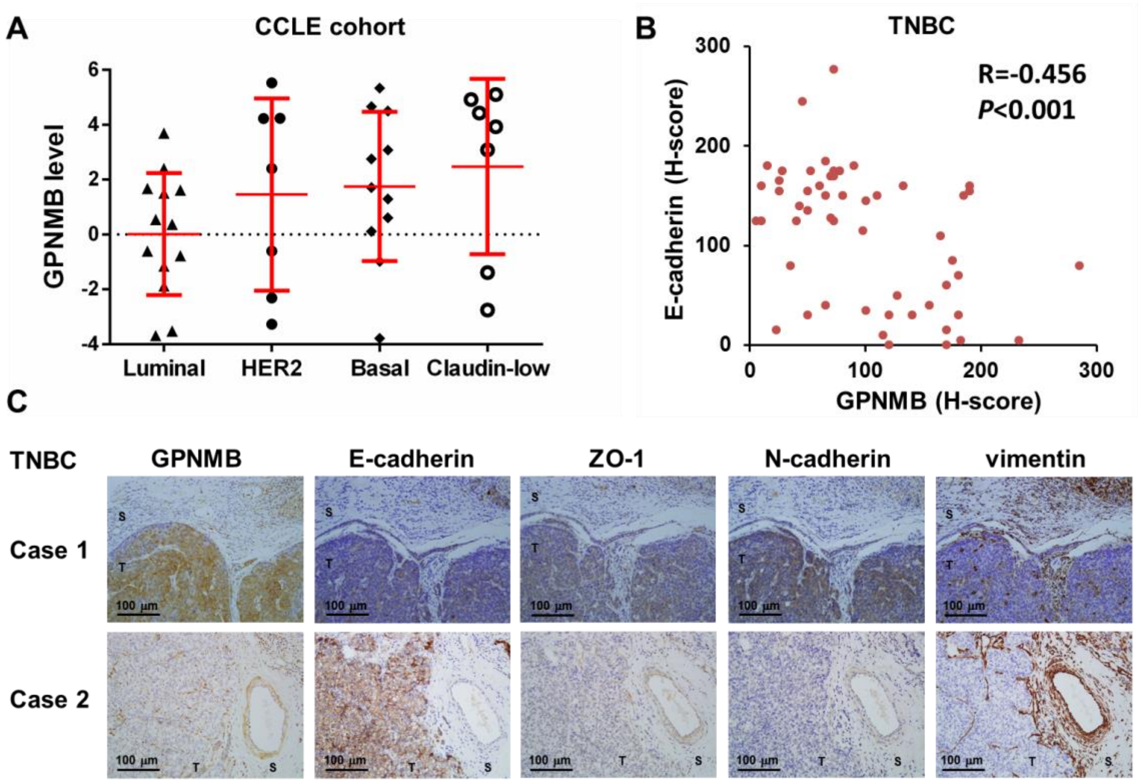

Figure S2. GPNMB protein level is negatively correlated with E-cadherin protein level in TNBC patients

(A) The GPNMB expression level compared in different subtypes based on gene expression from Cancer Cell Line Encyclopedia (http://www.broadinstitute.org/ccle, CCLE). (B) The correlation of between GPNMB and E-cadherin was analyzed. R, Pearson correlation coefficient. (C) The representative graphs of reciprocal expressions of GPNMB and EMT markers. T, tumor cells; S, stroma compartment.

Full-length blot

Full-length blot of Fig 4A. Whole-cell extracts of MCF 10A epithelial cells and TNBC cell lines were examined by Western blot analysis.

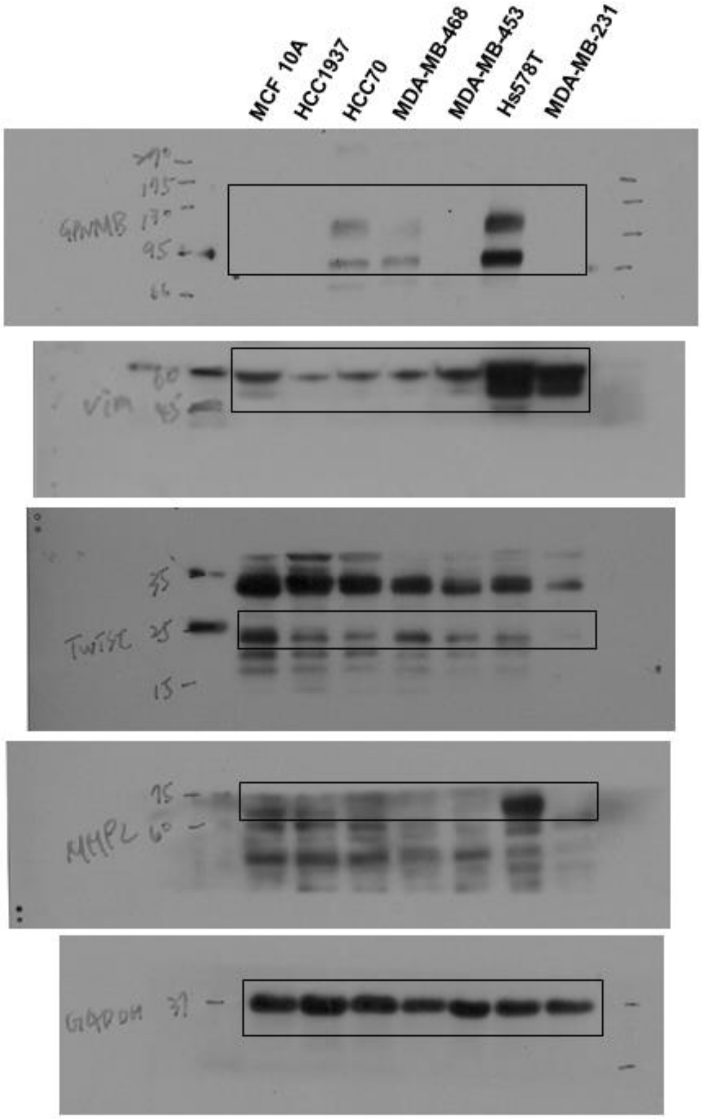

Full-length blot of Fig 4B. MDA-MB-468 and HCC1937 cells transfected with GPNMB expression construct or control plasmids (pCMV6) for 48 h were analyzed by Western blot analysis.

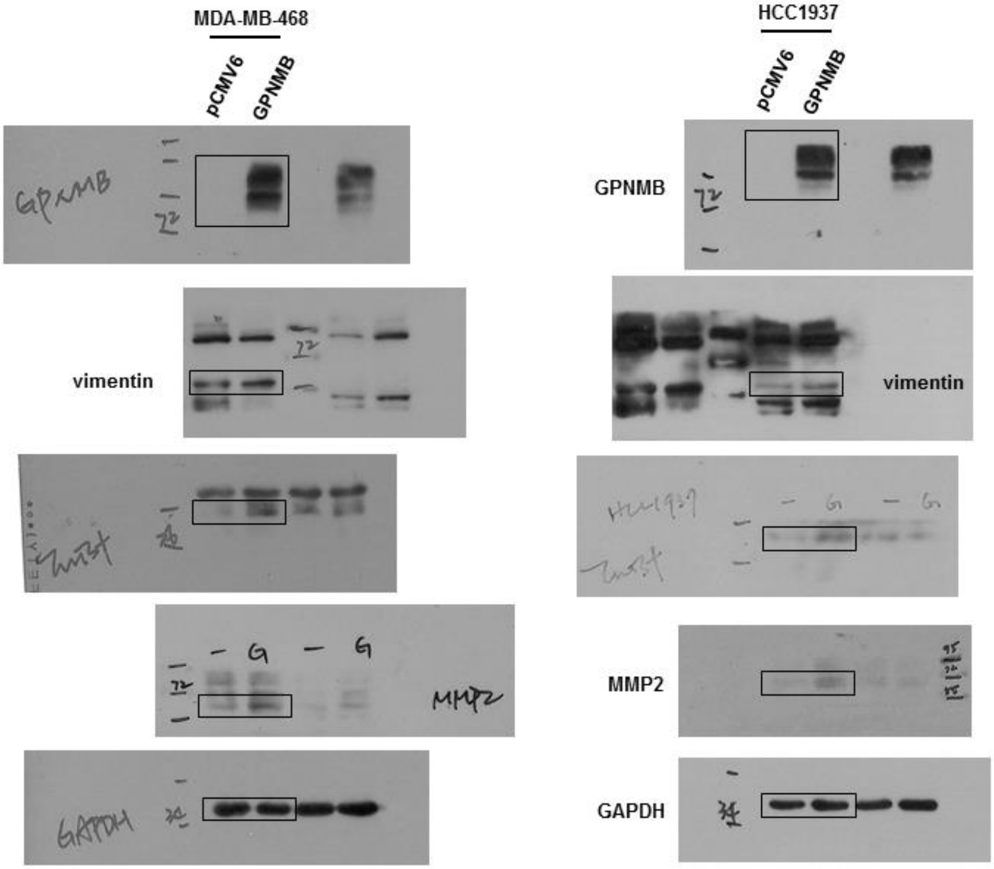

Supplement: Supplementary file 1 — Supplementary Information. [file 41598_2021_91588_MOESM1_ESM.pdf]
